# Demystifying MR Neurography of the Lumbosacral Plexus: From Protocols to Pathologies

**DOI:** 10.1155/2018/9608947

**Published:** 2018-01-31

**Authors:** Francisco J. Muniz Neto, Eduardo N. Kihara Filho, Frederico C. Miranda, Laercio A. Rosemberg, Durval C. B. Santos, Atul K. Taneja

**Affiliations:** Musculoskeletal Radiology Division, Imaging Department, Hospital Israelita Albert Einstein, São Paulo, SP, Brazil

## Abstract

Magnetic resonance neurography is a high-resolution imaging technique that allows evaluating different neurological pathologies in correlation to clinical and the electrophysiological data. The aim of this article is to present a review on the anatomy of the lumbosacral plexus nerves, along with imaging protocols, interpretation pitfalls, and most common pathologies that should be recognized by the radiologist: traumatic, iatrogenic, entrapment, tumoral, infectious, and inflammatory conditions. An extensive series of clinical and imaging cases is presented to illustrate key-points throughout the article.

## 1. Introduction

The lumbosacral plexus represents an intricate network of nerve unifications and divisions that results in terminal nerves responsible for sensory and motor innervation of the pelvis and the lower extremities [1]. Magnetic resonance imaging (MRI) of the peripheral nervous system has been performed since the 1980s and high-resolution neurographic sequences appeared in the 1990s [2].

In the last two decades, peripheral nerve evaluation was limited from a technical standpoint. Recently, technical advances in MRI and particularly with the advent of dedicated high-resolution magnetic resonance neurography (MRN) have optimized such task. MRN is an extraordinary and revolutionary technique that meticulously evaluates peripheral nerve diseases, since it allows the visualization of the anatomic origin of the nerves, their trunks course, and their path towards the lower limbs. MRN uses superior resolution imaging with the principles of highly weighted fat-suppressed T2 or STIR sequences, large-FOV, three-dimensional sequences, and postcontrast and diffusion-based MR imaging [3].

There are several clinical conditions that may require MRI of the lumbosacral plexus (Table 1). A wide range of etiologies may be involved in neuropathies: compressions, stretching, penetrating injuries, iatrogenic insults, frictions, entrapments, inflammation, metabolic, toxic, radiation conditions, and tumor diseases [4]. Clinical findings are sometimes confusing with lack of a specific diagnosis of the affected neural segment, or to detect involvement of multiple nerves, as seen in lumbar plexopathy. Besides that, common clinical conditions coexist, such as lumbar discogenic pain, hip osteoarthritis, and diabetes.

Historically, evaluation of peripheral neuropathies relied exclusively on neurophysiology and clinical examination to determine the exact location of the pathology. Electromyography (EMG) studies have limitations, mostly related to patient pain, nonspecific results in almost 1/3 of cases, and limited information on location, extent, and etiology of the nerve injury. Although they have high sensitivity, they lack specificity and displaying the anatomic detail needed to localize the nerve lesion and treatment planning.

Recently, MRN has been performed with higher magnetic field strengths (1.5 or 3.0-T) and high-resolution multiplanar sequences. MRN can directly assess nerve pathologies based on size and signal intensity changes, or indirectly by signs of muscle denervation [5]. This article aims to review MRN as a highly precise method to study peripheral nerves, describing anatomy, protocols, applications, pitfalls, and pathologies involved, along with an extensive case presentation and literature review.

## 2. Anatomy and Function

### 2.1. General Aspects

**Figure 1:**
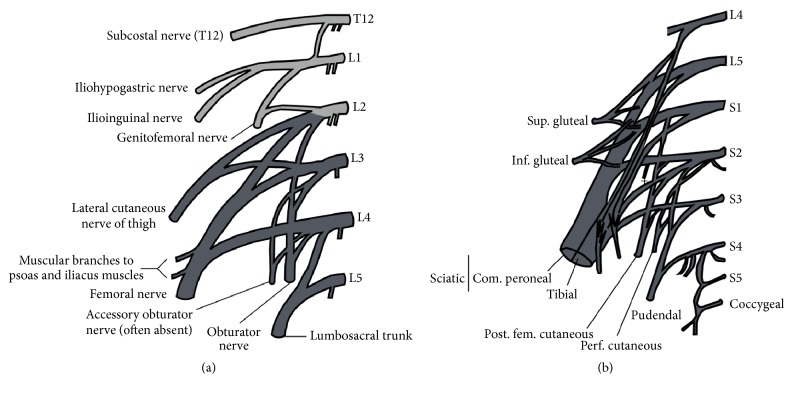
Illustrations ((a) and (b)) showing the anatomy of the lumbar plexus, which is formed by the ventral rami of L1–L4 ± T12, gathering inside or posterior to the psoas muscle belly. The lumbar plexus originates from the iliohypogastric nerves (T12 ± L1), ilioinguinal (L1 ± T12), genitofemoral (L1 and L2), femoral (L2–L4), cutaneous femoral (L2 and L3), obturator (L2–L4), and lumbosacral trunk (L4 and L5).  ^*∗*^Illustrations by FJMN.

**Figure 2:**
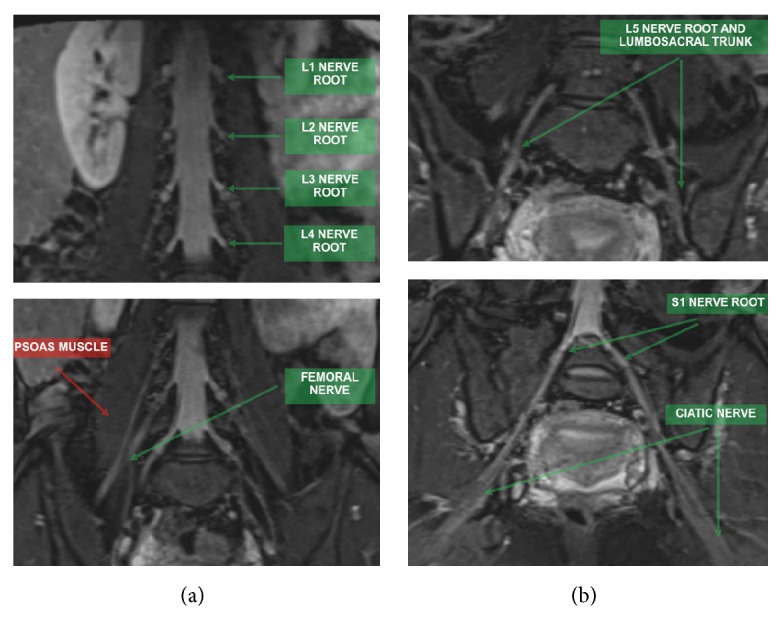
MR images in coronal STIR 3D SPACE (MIP) in (a), showing nerve roots of the lumbar plexus (L1 to L4) and the femoral nerve, formed by posterior nerve roots from L2 to L4, emerging lateral to the psoas muscle. In (b), MR images show L5 and S1 nerve roots, the lumbosacral trunk formed by the anastomotic branches of L4 and L5 nerve roots, and the sciatic nerve formed by major branches of the sacral plexus from L4 to S3.

The lumbar plexus is formed by the ventral rami of L1, L2, L3, and L4 nerve roots from the level of the L2 through L5 transverse processes, while the sacral plexus includes the ventral rami of L4, L5, S1, S2, and S3 nerve roots (Figures 1 and 2). Both together form the lumbosacral plexus, with the main role of innervating the lower limbs. The connection between these plexuses is made by the lumbosacral trunk (formed by L5 and smaller branches of L4 nerve roots). The lumbosacral trunk descends over the sacral wing and joins S1 to S3 nerve roots on the anterior aspect of the piriformis muscle to form the sacral plexus [6, 7].

The muscular landmark for the lumbar plexus is the psoas muscle, being the neural network connection formed within or posterior to it. Therefore, nerves can be classified according to their exit in relation to the psoas muscle margins, as follows:

- Medial to psoas: obturator nerve and lumbosacral trunk
- Lateral to psoas: iliohypogastric, ilioinguinal, genitofemoral, femoral, and lateral femoral cutaneous nerves.

### 2.2. Specific Nerve Segments (Table 2)

#### 2.2.1. Sciatic Never (Sciatic Plexus)

The sciatic nerve is formed by the ventral rami of the L4 to S3 nerve roots and is the largest peripheral nerve of the body due to its rich perineural fat, being easily assessed in all imaging planes on MRN [8]. Later it divides in tibial nerve, comum peroneal nerve, and posterior femoral cutaneous nerve.

The sciatic nerve may descend anteriorly, above, or within the piriformis muscle, with the anterior course being the most common. The sciatic nerve exits in the pelvis through the greater sciatic foramen, below the inferior margin of the piriformis muscle, and curves around the ischial spine and descends laterally to the common hamstring tendons and posteriorly to the external rotators of the hip as tibial and peroneal divisions (enclosed in a common sheath). In the thigh, it courses between the adductor magnus and the hamstring muscles. The sciatic nerve then divides in tibial (medial) and common peroneal (lateral) trunks in the distal third of the thigh [9].

The sciatic nerve has both motor and sensory functions:

- Motor innervation: to the posterior thigh muscles and all motor function below the knee (anterior, lateral, posterior, and deep muscular compartments). The tibial nerve provides motor function to the posterior compartment of the leg and plantar muscles. The peroneal nerve provides motor function to the anterior and lateral compartments of the leg.
- Sensory innervation: to the lower limbs excluding the medial sensory innervation of the tight and leg, which is provided by the obturator and femoral nerves [10].

#### 2.2.2. Lateral Femoral Cutaneous Nerve (LFCN)

**Figure 3:**
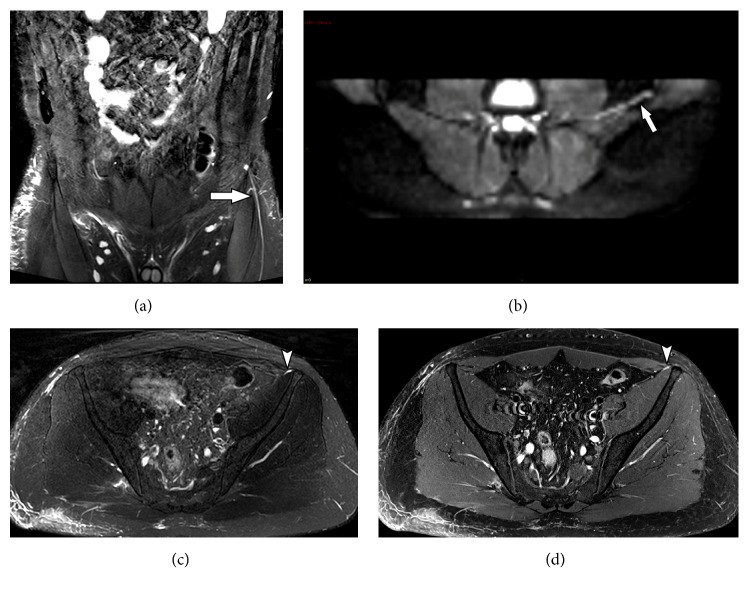
34-year-old male with history of thigh pain 1 month ago. MRN images in coronal T2 (a), axial diffusion-weighted imaging (b), axial T2 fat suppression (c), and T1 postcontrast (d) show signal change with slight thickening and contrast enhancement of the left lateral cutaneous nerve (solid arrow), predominating at the level of the fibrous tunnel underlying the inguinal ligament and iliac crest (arrowhead), representing neuritis.

The LFCN arises from the posterior divisions of L2 and L3 nerve roots. It crosses the lateral aspect of the psoas major and iliacus muscles and lies immediately medially to the anterior superior iliac spine (ASIS). The nerve may run below, above, or through the inguinal ligament in approximately 1.0 cm medially to the ASIS (Figure 3). The nerve abruptly turns down vertically to the subcutaneous tissue at the level of the inguinal ligament, coursing towards the upper thigh above the sartorius muscle [11]. Therefore, the anterior subcutaneous tissues must be included on sagittal and coronal images during MRN acquisition [12].

In the tight, it courses below the* fascia lata* and before transfixing its lateral aspect, it divides in anterior and posterior branches. These branches usually connect to the cutaneous branches of the femoral and saphenous nerves, forming the patellar plexus around the knee.

On T2-weighted MRN sequences, LFCN is isointense to the anterior surface of the iliopsoas muscle. Caution should be made while evaluating the segment that it courses across the inguinal ligament, where magic angle artifact may occur [13].

The LFCN has only sensory function:

- Motor innervation: none
- Sensory innervation: the skin of the anterior and lateral tight.

#### 2.2.3. Superior Gluteal Nerve

The superior gluteal nerve is formed by the posterior rami of L4, L5, and S1 nerve roots. It exits the pelvis through the great sciatic foramen just above the piriformis muscle, along with the superior gluteal artery and vein. Before it divides in superior and inferior branches, it can be seen in the fat-filled layer between* gluteus minimus* and* gluteus medius* muscles, and its final branches course inside* gluteus minimus* [14].

The best planes to assess the superior gluteal nerve in MRN are the coronal and sagittal, as it exits the pelvis close to the bony rim of suprapiriformis foramen, while in axial plane it is seen through the fat plane mentioned above. Due to their small size, gluteal nerves branches are not usually visualized by MRN, unless they are abnormally enlarged [15].

The superior gluteal nerve has only motor function:

- Motor innervation: abduct the tight at the hip by proving motor innervation to the gluteus minimum, gluteus medium, and tensor fascia lata muscles.
- Sensory innervation: none.

#### 2.2.4. Inferior Gluteal Nerve

The inferior gluteal nerve is formed by the posterior rami of L5, S1, and S2 nerve roots. It exits the pelvis through the sciatic notch, coursing below the piriformis muscle. At the lower border of the piriformis muscle, the inferior gluteal nerve turns backward and splits into superior and inferior branches, while it enters the* gluteus maximus* muscle. The inferior gluteal nerve lies medially to sciatic nerve. Unless it is abnormally increased, its small volume does not usually permits its identification by MRN [16].

Similar to the superior gluteal nerve, the inferior gluteal nerve also has only motor function:

- Motor innervation: gluteus maximus (acts extending the thigh)
- Sensory innervation: none.

#### 2.2.5. Femoral Nerve

The femoral nerve originates from the posterior rami of the L2, L3, and L4 nerve roots. It is the largest branch of the lumbar plexus. In the pelvis, the nerve courses downwards between the iliacus and psoas muscles and immediately lateral to the femoral artery and vein. The nerve exits from the pelvis below the inguinal ligament in a fibromuscular channel that includes the inguinal ligament, the iliopsoas, and the iliopectineal fascia [17].

In the proximal tight, the nerve enters the femoral canal lateral to the femoral vessels, and before it splits into anterior and posterior divisions, the femoral nerve originates a motor branch to the iliacus and the psoas muscles. The anterior division innervates the pectineus and sartorius muscles, while the posterior division innervates the quadriceps and originates the saphenous nerve.

In MRN sequences, the femoral nerve shows hyperintensity in T2WI at the level of the iliopsoas crotch and becomes isointense at the site of the inguinal ligament. On axial images, it may be seen as an area of subtle serration along the iliopsoas muscle surface [18].

The femoral nerve has both motor and sensory functions:

- Motor innervation: iliacus, psoas, pectineus, sartorius, and quadriceps muscles (acting in hip flexion and knee extension)
- Sensory innervation: medial thigh, anteromedial knee, medial leg, and foot (by the saphenous nerve).

#### 2.2.6. Obturator Nerve

The obturator nerve is formed by the ventral rami of the L2, L3, and L4 nerve roots. In the pelvis, if assessed in the coronal plane, the nerve courses along the psoas major medially and runs closely lateral to the sacrum and then exits the pelvis through the obturator canal (at the superior aspect of the obturator foramen).

Inside the pelvis, the nerve has a near vertical orientation just anterior to the psoas muscle (best seen on coronal plane in MRN). Within the obturator canal (along with the obturator vessels), it divides into anterior and posterior branches. The anterior branch supplies the hip and courses within a thin plane of fat between the pectineus and the obturator externus muscles. The posterior branch courses within the obturator externus muscle and the distal course is between adductor brevis and adductor magnus muscle [5].

In approximately 10% of subjects, there is an accessory obturator nerve arising from L3 and L4 nerve roots, which exits the psoas medially and courses parallel to the posterior division of the obturator nerve [3, 19], creating a communication with the femoral nerve. Injuries to obturator nerve are usually associated with femoral nerve injuries.

As seen in sciatic nerve, the more prominent perineural fat facilitates the identification of the nerve in all imaging planes by MRN.

The obturator nerve has both motor and sensory functions:

- Motor innervation:
  - Anterior branch: hip, gracilis, adductor brevis and longus muscles and sometimes the pectineus muscle
  - Posterior branch: obturator externus and adductor magnus muscles
- Sensory innervation: medial thigh and knee.

#### 2.2.7. Pudendal Nerve

The pudendal nerve is formed by the ventral rami of S2 and all of the rami of S3 and S4 nerve roots. The nerve is located between the piriformis and coccygeus muscles and exits the pelvis through the greater sciatic foramen.

After crossing the ischial spine, the pudendal nerve reenters the pelvis through the lesser sciatic foramen and hook around the sacrospinous ligament, just underneath the sacrotuberous ligament; these two ligaments outline an anatomic space that serves as reference to locate the nerve. The pudendal nerve courses inside the Alcock's canal, also known as Pudendal canal (along the lateral wall of the ischiorectal fossa). The Alcock's canal is formed by a division in the obturator fascia on the lateral wall of the ischioanal fossa [20].

The most important branch is the inferior rectal nerve that crosses the ischioanal fossa towards the anal canal and the external anal sphincter. The pudendal nerve also splits in perineal nerve and dorsal nerve of the penis and clitoris. Along its full course, the nerve is accompanied by the internal pudendal artery and a venous plexus (known as the pudendal neurovascular bundle).

Specially for the pudendal nerve, MRN aims to exclude lesions along its course, such as fibrosis, and serves to guide perineural injections [15].

The obturator nerve has both motor and sensory functions:

- Motor innervation: bulbospongiosus and ischiocavernosus muscles, as well as external urethral and rectal sphincters muscles
- Sensory innervation: perineum, scrotum, and anal muscles.

#### 2.2.8. Iliohypogastric Nerve

The iliohypogastric nerve is formed by the anterior division of L1 (mainly) and T12 (small contribution) nerve roots. The nerve courses along the lateral border of the psoas major and quadratus lumborum muscles, passing through the abdominal transverse muscles and lateral abdominal wall (above the iliac crest). It divides into anterior and lateral cutaneous branches [10].

The best sequence to assess the iliohypogastric nerve is the 3D MIP STIR SPACE at the level that it exits the psoas muscles.

The iliohypogastric nerve has both motor and sensory functions:

- Motor innervation: abdominal wall musculature
- Sensory innervation: skin above the inguinal ligament and superior lateral gluteal region.

#### 2.2.9. Ilioinguinal Nerve

The ilioinguinal nerve is formed by the anterior division of L1 (mainly) and the T12 (small contribution). Similar to the iliohypogastric nerve, the ilioinguinal nerve also courses inferiorly along the quadratus lumborum muscle and then enters the lateral abdominal wall medially to the inguinal ligament [21, 22].

Also, the best sequence to assess the ilioinguinal nerve is the 3D MIP STIR SPACE at the level that it exits the psoas muscles.

The ilioinguinal nerve has both motor and sensory functions:

- Motor innervation: abdominal wall musculature
- Sensory innervation: pubic symphysis, labia majora, femoral triangle, root of penis, and scrotum.

#### 2.2.10. Genitofemoral Nerve

The genitofemoral nerve is formed by the anterior division of L1 and L2 nerve roots. The nerve enters the psoas major muscle at the level of L3 and L4. It is divided in medial and lateral branches. The medial genitalis (external spermatic) in men enters the inguinal canal coursing along the spermatic cord; in women it courses along with the round ligament. The lateral (femoral branch) courses laterally do the femoral artery and posteriorly to the inguinal ligament, entering the sartorius towards the proximal thigh [23].

The genitofemoral nerve has only sensory function:

- Motor innervation: none
- Sensory innervation:
  - The lateral genitalis: proximal lateral aspect of the femoral triangle
  - The medial genitalis:
    1. In men: cremaster muscle, spermatic cord, scrotum, and adjacent thigh (responsible for the cremasteric reflex).
    2. In woman: the labia majora and adjacent thigh.

## 3. Protocols

High-resolution MRN requires tailored choice of the coil, pulse sequence, technique of fat suppression, slice thickness, and the field of view size (FOV) [8]. In order to identify abnormalities in fascicular morphology and signal intensity of the lumbosacral plexus components, parameters should have a balanced signal-to-noise ratio and adequate spatial-contrast resolution. In our institution, MR protocol for lumbosacral plexus is always performed at 3.0 T magnets (Siemens and GE), using a body surface coil (Table 3). The patient is positioned in dorsal decubitus with the limbs in full extension, with the MR coil encircling the lower abdomen and pubic region.

A complete evaluation of the lumbosacral plexus should include T1-weighted and fluid sensitive fat-suppressed sequence images [3, 19]: they serve as basis for MRN interpretation regarding peripheral nerve signal intensity (SI), course, caliber, fascicular pattern, size, and perineural fibrosis or mass lesions [24]. Our departmental protocol includes a total of eight sequences: sagittal STIR, sagittal T1 fast spin-echo, coronal STIR 3D SPACE, coronal PD 3D isotropic, Axial T1 fast spin-echo pre- and postcontrast, axial T2 SPAIR, axial diffusion, and coronal T1 3D VIBE pre- and postcontrast. Volumetric sequences (coronal STIR 3D SPACE, coronal T2 3D isotropic, and coronal T1 3D VIBE pre- and postcontrast) are fundamental for multiplanar (MPR) and maximum intensity projection (MIP) reconstructions. Diffusion-weighted sequence plays a key role in identifying earlier signal intensity abnormalities, also being reconstructed in MIP. The use of intravenous gadolinium-based contrast enhancement allows to asses inflammatory and infectious processes, tumor infiltration of the plexus, and posttraumatic neuromas.

MRN could be categorized as very heavy T2-weighted (T2W) sequences and diffusion techniques. T2-weighted MR sequence demonstrates the fascicular pattern of nerves. The DWI is useful to increase the accuracy due to suppression of vessel signal as well as offering the possibility to quantify apparent diffusion coefficient (ADC) values. This measurement may be useful to evaluate infiltrating tumors. Research protocols also include Diffusion Tensor Imaging (DTI), fractional anisotropy, and tractography [25, 26]. 3D sequences offer high spatial resolution for the study of nerve roots, perineural fat tissues, and the soft tissue around this structures. The T1W with thin slices and fat-suppression (FS) is useful to assess anatomical landmarks of the nerves by their contrast to the perineural fat tissues. High-resolution FS T2W sequences allow the visualization of changes in signal and thickness of the nerves [5, 9, 10]. Moreover, T1W and T2W images should be read simultaneously to explore signs of denervation (Table 4).

Based on the experience of the literature [3], there is less than 5% incidence of technical failure rate during high-resolution MRN acquisition. The main causes of failure include poor shimming, motion artifacts, field inhomogeneity, and susceptibility from nearby metal.

## 4. Interpretation and Pitfalls

MRN is a sensitive modality to characterize the plexus and peripheral nerve abnormalities, as it is able to demonstrate a lesion not detected by routine MRI. MRN findings in nerve disease are the rupture or distortion of normal fascicular morphology in T1-weighted images and neural hyperintensity in T2-weighted images. The abnormal high signal in nerve fascicles shows good correlation with clinical and electrodiagnostic evidence of nerve damage [27].

MRN is susceptible to pitfalls and artifacts that may lead to misinterpretation (Table 5). Here is a list of the main MRN-related pitfalls:

1. The* magic angle effect* (MA) is a potential artifact mimicking hyperintense SI in a normal peripheral nerve, which may lead to false-positive abnormalities by increasing intraneural T2 signal intensity [28]. That might happen when imaging a peripheral nerve in a plane higher than 55 degrees to the main vector of the magnet (*B*_0_). Intriguingly, different from tendons, MA can persist in echo time superior to 66 milliseconds and, therefore, to overcome such issue, higher TE values must be used [5]. However, the literature advises that the potential of the MA to cause the false-positive detection is low, even if the maximum MA occurs at 55° relative to *B*_0_, since a true neurological lesion should be discernable due to its stronger intraneural T2-WI hyperintense contrast [28].
2. The* hyperintense vascular signal* can also be a source of pitfalls. Despite the use of sequences with higher TE, small nerves can be interpreted as vessels when coursing along neurovascular bundles [29].
3. Sometimes,* fat suppression* of the images may not be homogeneous, especially in the pelvis, making the neural signal intensity confusing. This is due to the selection of wider field of view (FOV) in such protocols, as well as the widespread use of hip prostheses, which makes it even more difficult to optimize the acquisition protocol [30].
4. Increased* susceptibility and chemical shift artifacts,* usually seen at 3.0 T scans, may be counteracted by shortening the TE, performing parallel imaging, and increasing the bandwidth [31]. Moreover, 1.5 T MR imaging may be preferable to evaluate nerves in close proximity to a metal prosthesis.

## 5. Pathologies

### 5.1. Traumatic Lesions

**Figure 4:**
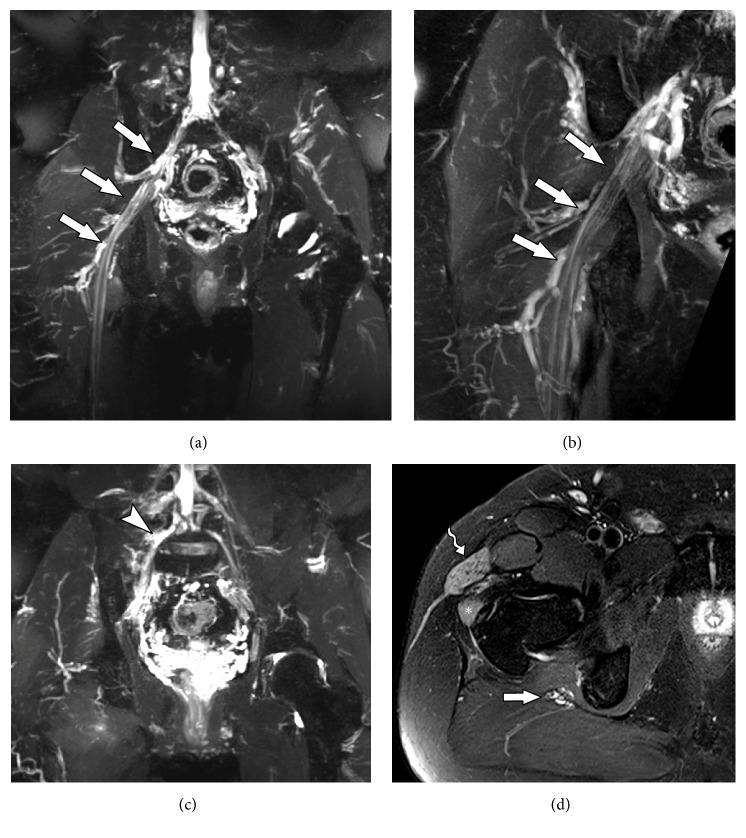
53-year-old male subject, with history of falling to the ground, presenting weakness in the right lower limb and with drop foot on physical examination. MRN images in coronal STIR 3D SPACE (a) and coronal T2 with fat suppression (b) of the right hip show slight thickening and elevation of signal intensity in the sciatic nerve (solid arrows) from its origin in the lumbosacral plexus to the thigh (neural stretching). MRN images in coronal STIR 3D SPACE (c) demonstrate lumbar plexus trunk thickening (arrowhead). MRN images in axial T2 (d) show diffuse edema affecting the tensor of fascia lata muscle (wavy arrow) and gluteus minimus (star), compatible with acute denervation/neuropathy.

**Figure 5:**
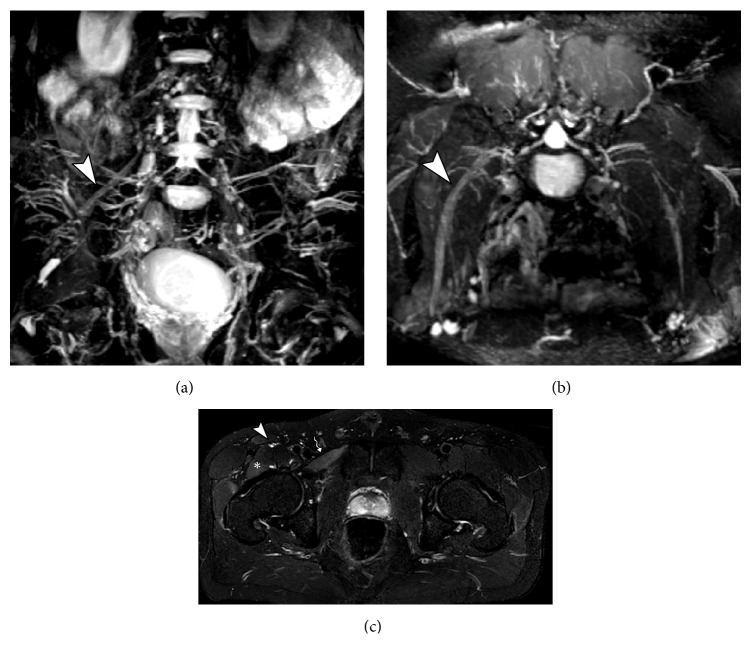
34-year-old male with history of chronic pain after traumatic injury in motorcycle accident for 2 months with surgery on the femur with plaque placement. MRN images in coronal STIR 3D SPACE ((a) and (b)) and axial T2 fat suppression (c) show signs of right femoral neuropathy, with thickening and elevation of the femoral nerve signal in the retroperitoneal and proximal thigh trajectory (arrowhead). Early stage denervation is associated with edema and a slight contrast enhancement of the distal iliopsoas (star) and pectineus (wavy arrow) muscles.

Lesions to the plexus stand in the the most difficult and terrible scenarios of nerve injuries, due to the difficult recovery of function and pain in lower extremities [32]. The first clear description of a lumbosacral plexus injury came only approximately 50 years ago [33]. Opposing to the brachial plexus, the lumbosacral plexus has the supportive strength of the bony pelvis, which protects the nerve roots against excessive stretching and rupture [34]. In the brachial plexus injuries, the main mechanism is direct trauma, while in the lumbosacral plexus it is indirect traumas (Figures 4 and 5), as well as spinal-related injuries, hip dislocations and fractures, inguinal hernia, aortic aneurysms, and lesions affecting psoas muscle (such as hematomas and abscesses). In severe pelvic fractures, one can usually find sacroiliac joint dislocations and traction lesions to the lumbosacral plexus and spinal nerves or* cauda equina* roots.

In order to manage the best therapeutic choice in traumatic lesions of the peripheral nerves, it is of major importance to know what type of injury that occurred, since some lesions are self-limited and have potential for regeneration and spontaneous regression. On the other hand, for the treatment of more serious lesions, surgical procedures may be necessary [35].

The Seddon classification (1943) divided peripheral nerve lesions in neuropraxia, axonotmesis, and neurotmesis.* Neuropraxia* results from local compression or ischemia of the axons with focal demyelination; recovery is spontaneous and occurs within days or weeks. There is transient loss of fiber conductivity and Wallerian degeneration does not occur. In* axonotmesis*, there is interruption of axon continuity, but the connective tissue of sustentation is preserved or without significant injury; peripheral regeneration occurs slowly. There is Wallerian degeneration of the distal axon to the area of aggression and motor and/or complete sensory paralysis occurs. One key concept is the Wallerian degeneration (anterograde degeneration), which results from a nerve fiber injury causing death of the axon distal to the lesion (further away from the neuron's cellular body). The conduction of electrical stimuli is therefore lost. In* neurotmesis*, the nerve is completely sectioned or destroyed. The only hope for recovery is, in such cases, surgical repair with appropriate suture tissue [36].

The process of nerve regeneration and degeneration can be analyzed with neurography, along with diffusion and tractography [37]. The lack of recovery in traumatic lesions may lead to neuroma formation, which can be identified properly using MRN [24].

### 5.2. Iatrogenic Injuries

MRI of the lumbosacral plexus may demonstrate neural injuries within the immediate intraoperative period or even later in the stage of fibrosis, where it may cause root entrapment. This situation is commonly seen when symptoms persist after surgery.

Patients with history of pelvic tumors may be treated with radiotherapy and develop actinic plexopathy. Actinic abnormalities manifest with tissue fibrosis, presenting low signal in all MR sequences in the late phases but may present high signal with small postcontrast enhancement in the earlier stages. There is also neural architectural distortion and diffuse thickening of portions of the lumbosacral plexus, unlike metastatic lesions, which present as focal masses with contrast enhancement and might restrict in diffusion sequences [38]. Finally, other causes are nerve damage by alcohol and thermal ablation treatment.

### 5.3. Entrapment Lesions

Compression neuropathies may present with pain and functional deficit within the affected nerve territory. The clinical course may be vague and poorly localized on physical examination, making it difficult to distinguish from other causes of nonneurological pain.

In changes caused by chronic compression, nerve damage occurs progressively or intermittently, sometimes with periods of remission and exacerbation. As a result of recurrent trauma or chronic ischemia, fibrosis may involve the neural fascicles or the tissue around. In more severe cases, the nerve fibers may be destroyed and the neural trunk replaced by fibrotic tissue if surgical decompression is not performed.

Entrapment conditions to the lumbosacral plexus can be divided in spinal or extraspinal compression.

#### 5.3.1. Spinal Compression

Pathologies affecting the lumbosacral plexus may cause neuropathic pain (radiculopathy), being a factor of diagnostic challenge for the clinician and radiologist, due to the deep locations of the nerves and variable regional innervation [39]. Compression of spinal nerve roots may be identified by signal intensity abnormality as a result of direct mass effect. This can lead to changes in MRI pattern such as muscle denervation in respective myotomes. Advanced osteoarthritic processes of the spine associated with scoliosis are one of the common causes of root compression.

**Figure 6:**
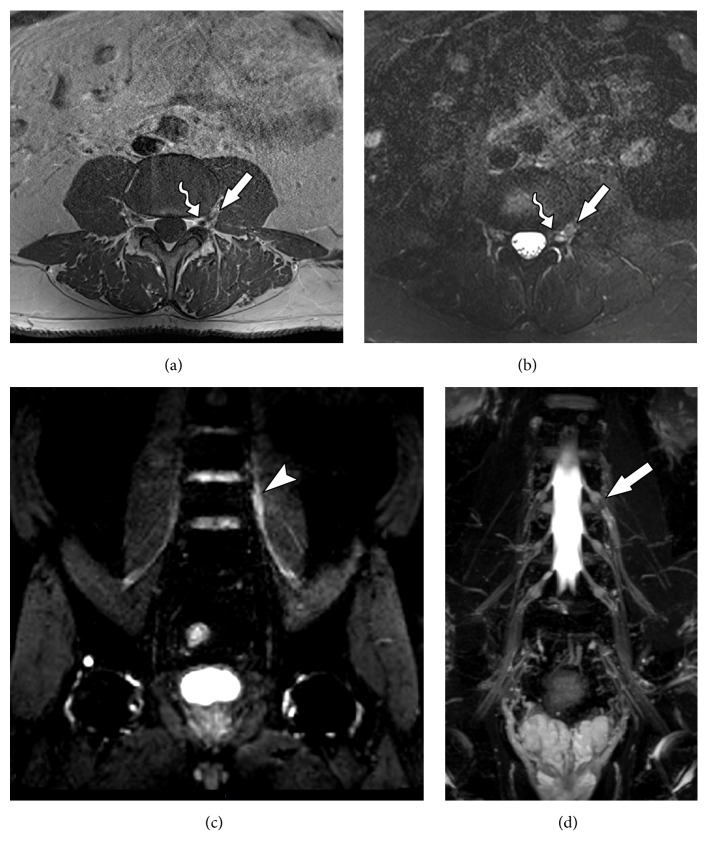
65-year-old male admitted to the emergency room with history of sudden low back pain radiating to the left lower limb. MRN images in axial T1 (a), axial T2 fat suppression (b), coronal diffusion-weighted imaging (c), and coronal diffusion-weighted imaging MPR reconstruction (d) show left foraminal/extreme lateral discal hernia in L3-L4 level, with extruded discal fragment (solid arrow) compressing the left L3 nerve root (wavy arrow) along its emergence. Slight thickening is associated in the proximal segment, as well as T2 hyperintensity and diffusion restriction (arrowhead).

**Figure 7:**
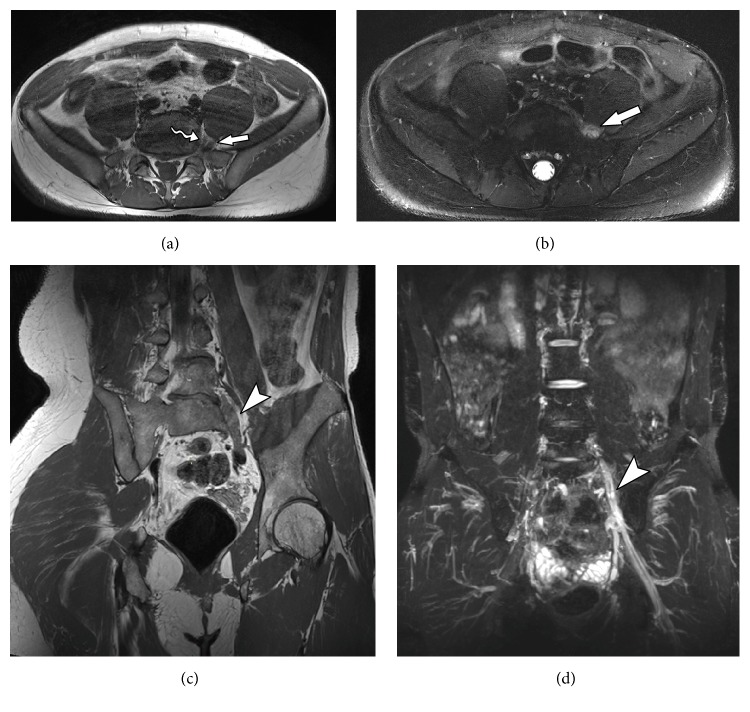
34-year-old male with history of left hip pain with radiation to lower left limb after soccer training. MRN images in axial T1 (a), axial T2 fat suppression (b), coronal T2 3D SPACE (c), and coronal STIR 3D SPACE (d) show a heterogeneous tissue (solid arrow) with high intensity in T2, with low signal foci in perineural region posterior to the left L5 nerve root (wavy arrow) anteriorly to the left sacral wing. There is thickening and abnormal signal intensity of the left nerve root of L5 (arrowhead), as well as of fascicles of the nerve to the proximal thigh segment, with a high T2 signal and postcontrast enhancement (neuropathy). The appearance is compatible with L5-S1 left extraforaminal disc extrusion material, with extensive lower migration and neural compression.

MRN can potentially identify spinal compression not detected by conventional spinal MRI, including entrapment in extraforaminal segment of the nerve root, such as a lateral herniated disc (Figures 6 and 7) or suboptimal decompression surgery. MRN provides targeted anatomical and lesion assessment, especially correlating to the symptoms. MRN is also useful to demonstrate anatomic variants in femoral and sciatic nerves, which, similar to what occurs to incidental discal herniation not impinging nerves, does not imply that it could cause symptoms. Direct visualization of neuromuscular abnormality is an indispensable finding [19].

#### 5.3.2. Extraspinal Compression

The lumbosacral plexus can be directly affected by extrinsic compression and diffuse infiltration or secondary to systemic diseases and inflammatory processes.

**Figure 8:**
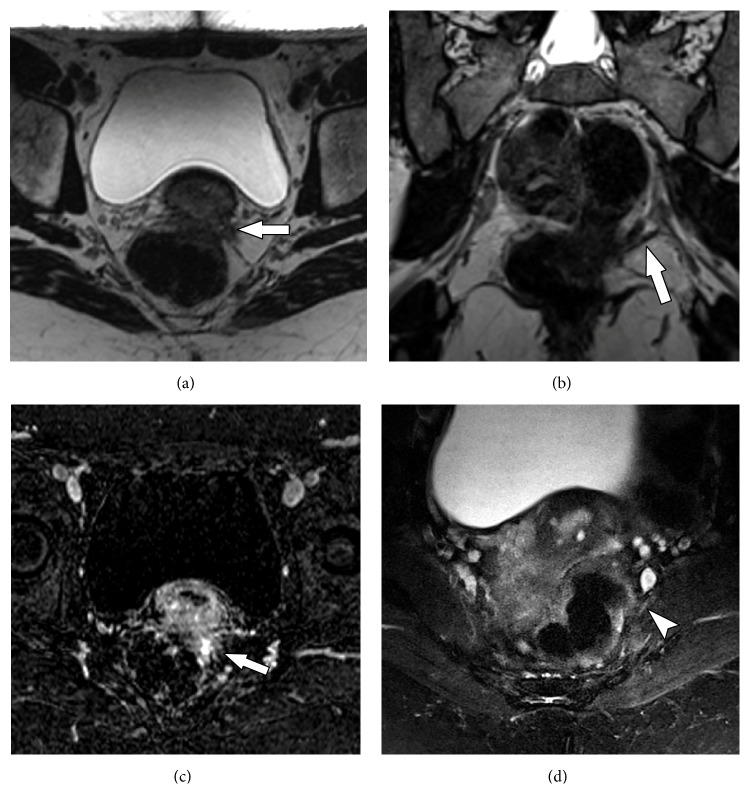
31-year-old female subject, presenting numbness and tingling on the left lower extremity for three months. Past medical history of endometriosis. MRN images on axial T2 isotropic (a), coronal T2 isotropic (b), axial T1 VIBE subtraction (c) show deep endometriosis in the uterine retrocervical region, extending to the left paracervical space maintaining an intimate relationship with the pelvic root path (solid arrows) and axial T2 FLAIR (d) demonstrates slight change of the emerging left S2 root signal intensity with mild contrast enhancement (arrowhead).

While pelvic pathologies affect primarily the sacral plexus, many intra-abdominal pathologies can damage the lumbar plexus, especially due to the involvement of the psoas muscles in trauma or surgical interventions, hematomas associated with anticoagulant therapies, abscesses, and tumor infiltration, or related to endometriosis (Figure 8). There are anatomical regions in which segments of peripheral nerves are vulnerable or predisposed to become trapped and suffer from chronic compression. Neural compression occurs especially in osteofibrous tunnels but may also occur at points of passage of the peripheral nerve through the muscles or near a band of fibrous tissue.

Extraspinal compression occurs when the nerve signal or caliber changes adjacent to an anatomic structure that may cause mass effect such as bone, fibrous bands, or even accessory muscle belly.

One of the most common situations is the use of MRN to identify surgically treatable piriformis syndrome. Sciatica without evidence of lumbosacral root compression is sometimes attributed to piriformis syndrome [40]. In this setting, MRN can demonstrate sciatic nerve entrapment by the piriformis muscle or by an associated fibrous band, without the need for surgical exploration to identify the compression [41].

The identification of extraspinal entrapment sites is another useful indication of MRN as a result of planning for successful decompression or targeted injections [40].

### 5.4. Tumors of Lumbosacral Plexus

#### 5.4.1. Benign Tumors

**Figure 9:**
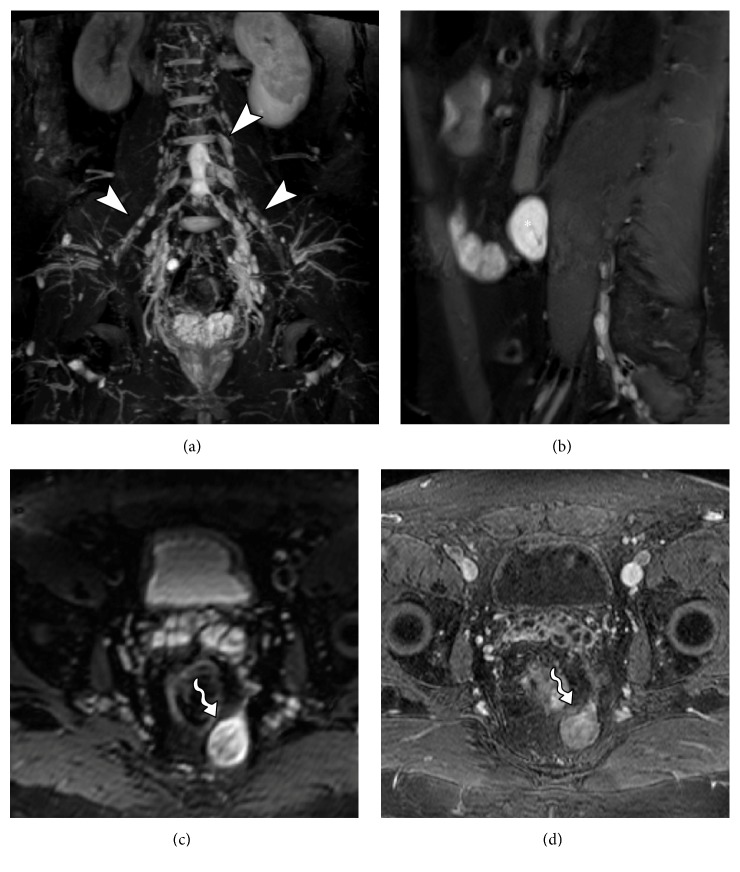
38-year-old male with history of neurofibromatosis with low back pain. MRN images in coronal STIR 3D SPACE MIP (a), sagittal STIR 3D SPACE (b), and axial STIR 3D SPACE (c) and axial T1 VIBE postcontrast show multiple small nodular images (arrowhead) in the neural roots of the lumbosacral plexus, with bilateral nodular thickening measuring up to 2 cm, extending from the emergencies to the sciatic nerves. Compatible with neurofibromas in the clinical setting. Two major nodular lesions with well-defined contours, one in the left lateral topography of the aortic bifurcation (star) and the other in left perirectal topography (wavy arrow) that may represent neurofibromas with degeneration.

Benign neural sheath tumors originating from a nerve are wrapped by the epineurium or perineurium, presenting a true capsule. The two major types of benign tumor of the neural sheath are schwannoma (or neurilemoma) and neurofibroma [42]. Although mostly solitary, multiple plexiform schwannomas are associated with Type 1 neurofibromatosis (NF-1). In large nerves, masses are generally eccentric and displace the neural fibers (Figure 9) [43].

Neurofibromas may present in three types, according to their distribution: localized, diffuse, and plexiform, the first being the most frequent form (90%) [44]. Diffuse neurofibromas are usually poorly delimited from subcutaneous fat tissue and infiltrates the connective tissue septa. They are associated with NF-1 in about 10% of cases.

Neurogenic tumors classically arise outside the central nervous system and include both typical (nonplexiform) and plexiform neurofibromas (NPX). They are benign and originate from the endoneurium [43]. Among clinical findings of NF-1, NPX alone is sufficient to define the diagnosis. It is important to stress that “plexiform” does not imply involvement of a neural plexus, since the term plexiform refers to its growth aspect similar to a network, involving multiple fascicles of the nerve, which becomes thickened and surrounded by a proteinaceous matrix, forming nodules or masses [45].

NPXs often involve nerve plexuses and dorsal roots, as well as other deep structures, mainly major nerves. The NPX is divided into superficial and deep to the fascia. They have different characteristics that can be recognized by imaging studies, observing that superficial type is generally unilateral, with infiltrating edges and diffuse pattern, while deep type tends to be bilateral, with defined borders and nodular pattern (“target appearance”). NPXs have a predilection for nerves about the spine and can progress slowly to distal segments. Signs of rapid growth and persistent pain call attention to possible malignancy [44].

#### 5.4.2. Malignant Tumors

*Malignant Schwannomas*. Malignant schwannomas present as large masses affecting the entire thickness of the nerve, with proximal and distal extension, and involving large nerve trunks. Malignant schwannomas, as well as neurogenic sarcoma and neurofibrosarcoma, may be de novo or arise from benign tumors. About 25–50% of malignant neural sheath tumors occur in patients with NF-1 [42]. In follow-up exams, some NPX may develop minor areas of malignant alterations, which can be hard to identify due to the large volume and growth of the lesion [45].

Usually, the differentiation between benign and malignant neural sheath tumors is not safe by imaging only. Some features favor malignancy, including large sizes (>5 cm in its maximum diameter), poorly defined margins, peripheral contrast enhancement, cystic or necrotic changes, highly heterogeneous intensity signal, substantial growth interval, infiltrative borders, and perilesional edema [46].

**Figure 10:**
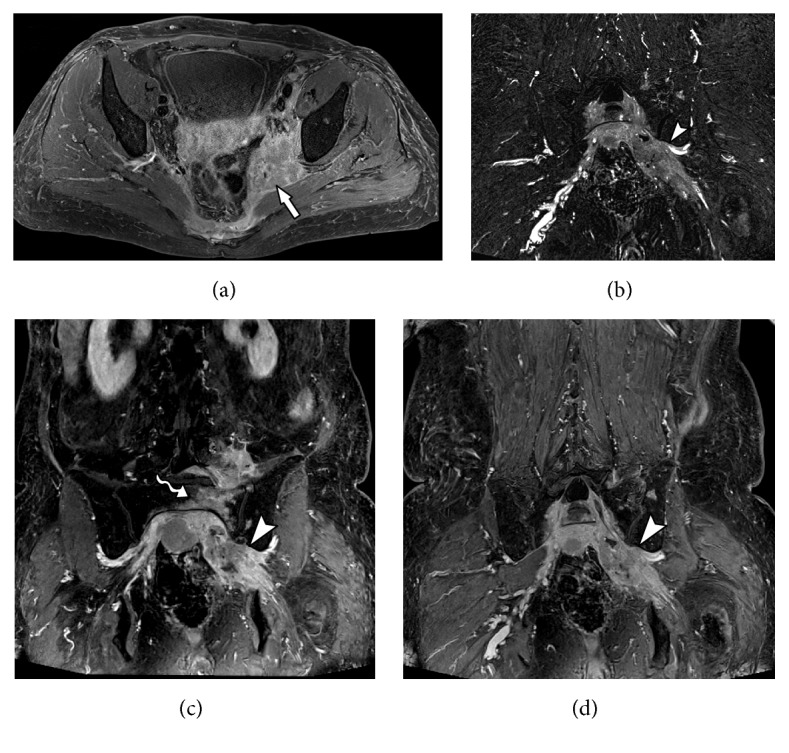
73-year-old female with pain for 4 months in sciatic territory, with history of Hodgkin lymphoma undergoing chemotherapy. MRN images in axial T1-weighted contrast-enhanced (a), coronal VIBE subtraction (b), and coronal T1 postcontrast VIBE 3D SPACE ((c) and (d)) show a large, lobular, contrasting, infiltrative mass (solid arrows) suggestive of a lymphoproliferative process, disseminated inferiorly through the left paravertebral region near the psoas and presacral region. The mass also infiltrates the left neural foramina and emergent nerve roots of L4-L5 and L5-S1 and, bilaterally, in S1-S2 to S4-S5. It determines diffuse involvement of the left sacral plexus, proximal segments of the obturator, pudendal and sciatic nerve, close to the piriformis and internal obturator muscles (arrowhead). There is also signs of bone infiltration (wavy arrow).

*Neurolymphomatosis. *Perineural lumbosacral plexus infiltration is an uncommon manifestation of a spread of systemic lymphoma and is therefore difficult to diagnose only based on conventional imaging techniques. Neurolymphomatosis (NL) has been described as an isolated entity or associated with systemic or primary CNS lymphoma (Figure 10). Differential diagnoses panels are extensive and include infection, malignant peripheral nerve sheath tumor, sarcoidosis, demyelinating and immune-mediated polyneuropathy, and hypertrophic neuropathy [27].

**Figure 11:**
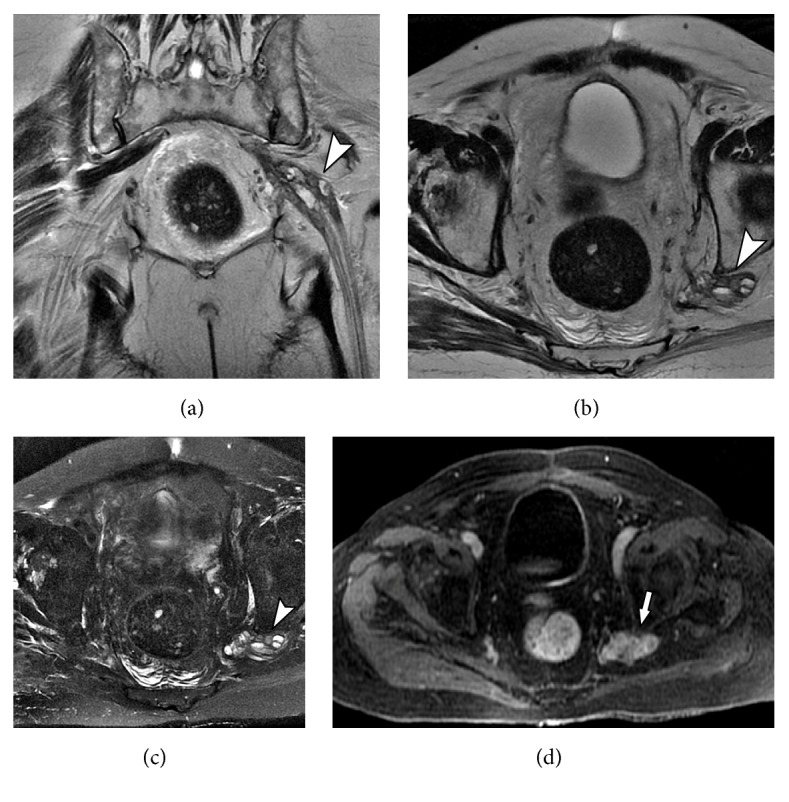
73-years old male with history of weakness and paresthesia in the lower left limb. Previous history of left nephrectomy due to renal tumor 5 years ago. MRN images on coronal T2 3D isotropic (a), axial T2 (b), axial T2 with fat suppression (c) and axial axial T1-weighted contrast-enhanced (d) showing a solid tissue and neural thickening of the proximal sciatic plexus, with contrast enhancement (solid arrow) and cystic areas of permeation (arrowhead), characterizing neoplastic infiltration.

**Figure 12:**
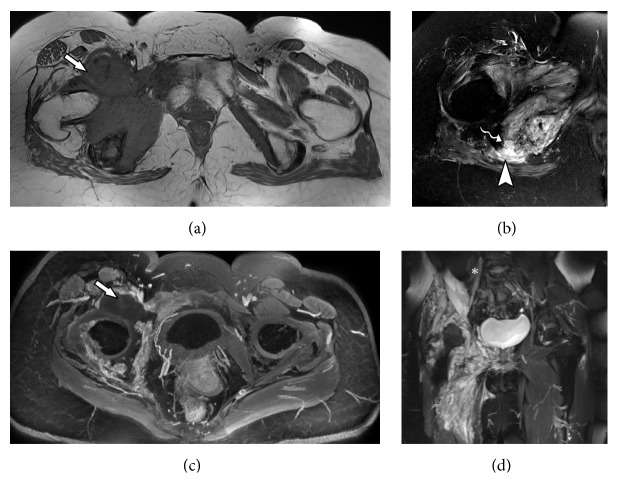
48-years female smoker with lung cancer developing lumbar and right limb pain. MRN images in axial T1 (a), axial T2 fat suppression (b), axial T1 VIBE post contrast (c) and coronal STIR 3D SPACE MIP (d) show a large right hip mass (solid arrow) with soft tissue components and necrosis associated with edema and enhancement of adjacent soft tissues (arrowhead). The inflammatory process involves the sciatic nerve (wavy arrow), with thickening and contrast enhancement. Femoral neuropathy is also seen (star).

**Figure 13:**
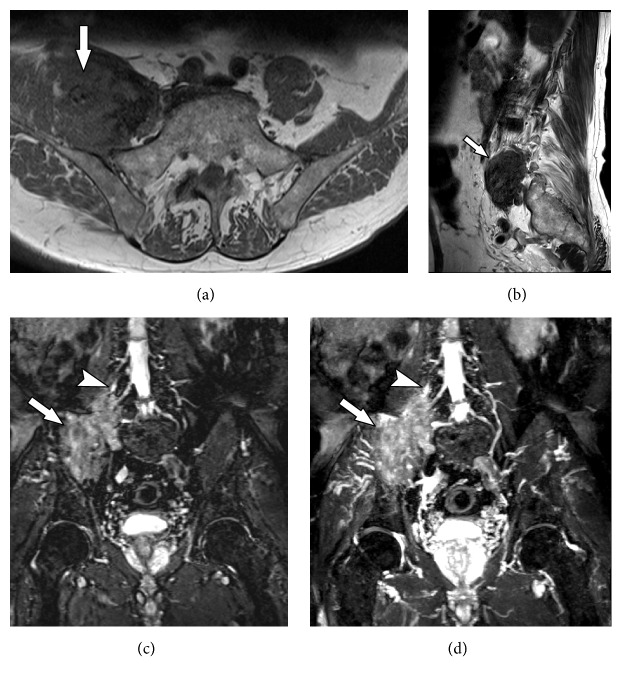
74-years male with low back pain related to a paravertebral tumor. Biopsy revealed an undifferentiated liposarcoma. MRN images in coronal axial T2 (a), sagittal T2 (b), coronal STIR 3D SPACE (c) and coronal STIR 3D SPACE MPR (d) show a heterogeneous solid mass with high T2-weighted signal intensity predominance and contrast enhancement, infiltrating the right psoas muscle in L5 and S1 planes (solid arrow). There is also an extension to the iliac musculature and right lateral aspect of L5 vertebral body, with small insinuation to the neural L5-S1 foramen. The lesion involves the extraforaminal pathway of the L4 root and the proximal portion of the femoral nerve (arrowhead).

*Secondary Tumors. *Secondary involvement of the lumbosacral plexus occurs by direct extension, contiguity, or lymphatic dissemination. Although rare, metastatic implants to the lumbosacral plexus usually origin from melanomas and less commonly from breast, lung, and kidney tumors (Figures 11 and 12). MRN can not differentiate between primary and secondary involvement, and knowledge based on previous clinical and histopathological records, involvement of adjacent lymph nodes and bones, or multisystemic conditions, along with infiltrating appearance of the lesions, is essential to favor such diagnosis (Figure 13) [9].

### 5.5. Infectious Plexopathy

The direct lumbosacral plexus injury secondary to systemic diseases is uncommon, although there are reports of plexopathy induced by varicella zoster virus, mycobacterium tuberculosis, HIV, and hepatitis C virus. Most of the available case reports do not present MRN images, being diagnosed based on neurological examination, conventional MRI, EMG, and laboratory examination results [47].

**Figure 14:**
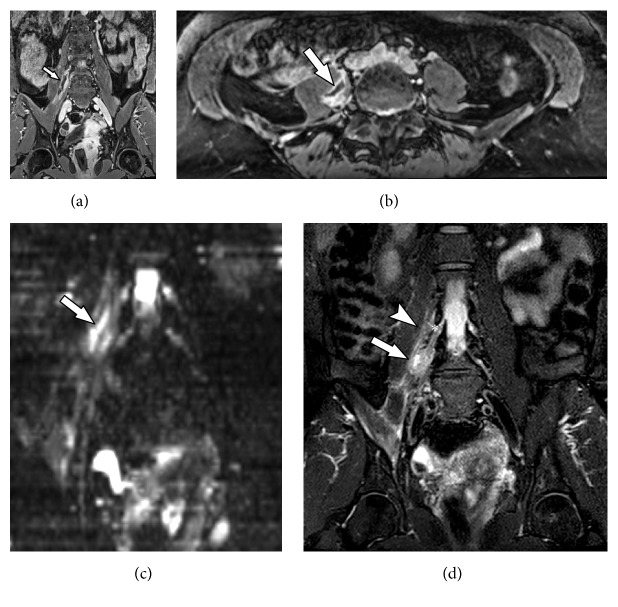
43-years male in late postoperative stage of gastroplasty with progressive low back pain and febrile spikes. MRN images in coronal T1 VIBE post contrast (a), axial T1 VIBE post contrast (b), coronal STIR coronal diffusion-weighted (c) and coronal STIR 3D SPACE (d) show fluid collection in the surgical bed at the right iliopsoas muscle, with intense peripheral contrast enhancement (solid arrow). The L3 nerve root (arrowhead) presents signs of neuropathy. The right L4 nerve root (star) emerges through the surgical bed and is also diffusely thickened, with edema and contrast enhancement throughout its course, up to the level of the inguinal region.

The special localization of the lumbosacral plexus in close contact with the psoas makes it susceptible to contiguous processes due to its own muscle affections (myositis) or adjacent retroperitoneal space. Thus abscesses in the psoas (Figure 14) or even in the gluteus region are causes of affection of the plexus [48].

It is worth mentioning that the infection can also be originated locally in an organ of the adjacent genitourinary or gastrointestinal tract, or due to an infectious process of the intervertebral disc as in the discitis. Diseases like tuberculosis, Crohn, or HIV can also determine the formation of perirectal abscesses.

A rare cause that must be remembered in tropical countries is an infection commonly seen in HIV patients causing a plexopathy known as diffuse infiltration lymphocytosis syndrome (DILS) [49].

### 5.6. Inflammatory Plexopathy

#### 5.6.1. Diabetic Plexopathy

**Figure 15:**
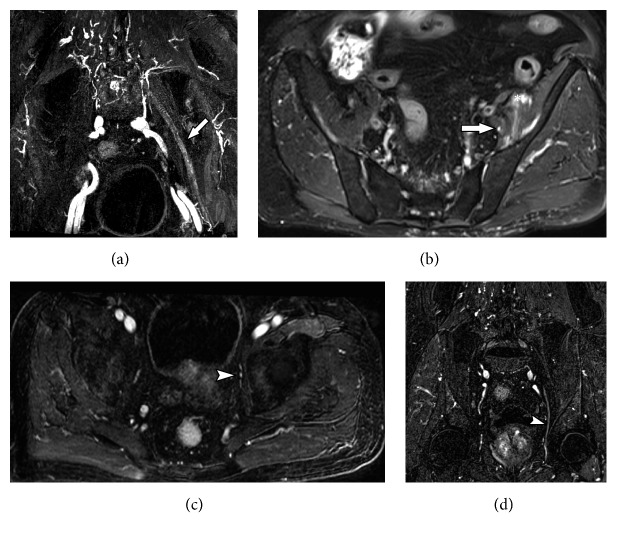
83-years male, diabetic patient non-responsive to insulin therapy, presenting chronic pain in the lower limbs. MRN images in coronal STIR 3D SPACE MPR (a), axial T2 fat suppression (b), axial T1 VIBE subtraction (c) and coronal T1 VIBE subtraction (d) show left femoral nerve (solid arrow) thickening with inflammatory signs, throughout from its origin to the thigh, associated to diffuse edema perineural planes. Fluid is seen around the muscle, and along the neural path (star). Obturator nerve (arrowhead) is discretely thickened and with inflammatory signals since its origin to distal portions. These findings are suggestive for inflammatory polyradiculoneuropathy.

Lumbosacral radiculoplexus neuropathy (LRPN) is an uncommon but distinct condition characterized by asymmetrical lower extremity pain, weakness, and muscle atrophy affecting commonly the thigh muscles; mild sensory symptoms are seen. The diabetic lumbosacral polyradiculoneuropathy (diabetic amyotrophy) represents an ischemic lesion with microvasculitis, demonstrating multifocal fiber loss, perineural thickening, neovascularization and neuromas associated with perivascular inflammatory fluid collections, vessel wall inflammation, and hemosiderin-loaded macrophages in histopathological analyzes (Figure 15) [50]. A high index of suspicion is important to this clinical diagnosis. Patients are often misdiagnosed as having compressive lumbosacral polyradiculopathy, which is supported by the “incidental” finding of spondylotic changes of the lumbosacral spine [51].

MRI of the lumbosacral spine and plexus are useful in ruling out other potential etiologies such as compressive polyradiculopathy, infiltrative plexopathy, or compression of the plexus by a hematoma or tumor mass [52]. At MRN, hyperintense T2 signal in nerves can be seen, as well as nerve root hypertrophy and postcontrast enhancement. Commonly, diabetic plexopathy comes along with muscular denervation, presenting hyperintensity in T2 in acute or subacute stages, and muscle atrophy with T1 hyperintensity due to fatty infiltration in chronic stages.

#### 5.6.2. Chronic Inflammatory Demyelinating Polyneuropathy

Chronic inflammatory demyelinating polyneuropathy (CIDP) is a rare autoimmune demyelinating neuropathy, usually responsive to steroids therapy [53]. It evolutes with progressive weakness and sensory impairment of the upper and lower limbs, associated with hyporeflexia or diffuse areflexia, sometimes with bouts of exacerbation and remission. Electrophysiological studies show signs of demyelination, with nerve conduction blockage (two or more nerves). Increased protein can be seen in cerebrospinal fluid analysis. Nerve biopsy may be necessary for the definite diagnosis.

On imaging, there may be thickening of the distal and/or proximal nerves, including thickening of nerve roots and structures of the brachial or lumbar plexus. The disease is closely related to* Guillain-Barré Syndrome* (GBS), in which inflammation and acute demyelination of peripheral nerves occurs. The symptoms of these two conditions are similar and they are distinguished by the progression pattern: in GBS, there is a rapid progression, and the most severe signs usually appear within the first four weeks [54].

In CPID, MRN shows fusiform hypertrophy of the peripheral nerves and the thickening of the nerve roots. Exceptionally, hypertrophy can be seen in* cauda equina *[55]. It is important to note that these hypertrophic changes are not exclusive to ICPD and may be also detected in hereditary Charcot-Marie-Tooth lymphoma and other polyneuropathies. Recently, MRN has been used to distinguish different subtypes of CIDP based on the pattern of nerve hypertrophy [56].

#### 5.6.3. Charcot-Marie-Tooth

Charcot-Marie-Tooth disease (CMT) encompasses a heterogeneous group of hereditary sensory and motor neuropathies that occur with hypertrophic peripheral neuropathy. The manifestation of the disease usually occurs in the first decades of life, with progressive distal weakness, absence or diminution of tendon reflexes, atrophy of peroneal muscles, and discrete sensory loss. Clinical findings include atrophy and sensory loss affecting the extremities, ataxia, areflexia, palpable tangled peripheral nerves, cavus feet, and hammer toes.

In MRI, there is hypertrophy and increased abnormal contrast enhancement of the nerve roots and peripheral nerves. Bilateral lumbosacral plexus and peripheral nerve involvement are common. The enlarged nerves are frequently related to increase in interfascicular epineurium with atrophic demyelinated fascicles [57].

#### 5.6.4. Idiopathic and Other Conditions

Other causes include drugs vasculitic plexopathy, mononeuritis multiplex, hepatitis C-associated vasculitis, sarcoidosis, neuro-Behcet's disease associated vasculitic plexitis, and amyloidosis involving the plexus. MRN is useful to localize painful sensory neuropathies to the dorsal root ganglion, even with normal electrical tests [58]. If the cause of the plexopathy is not diagnosed, MRN can identify hypertrophic neuropathies and monitor their pattern of increase to target the clinical workflow and therapy.

A summary for the MRN features of the above pathologies affecting the lumbosacral plexus is presented in Table 6.

## 6. Conclusions

With the improvement of the available technology, MRN should be appreciated to diagnose pathologies of the lumbosacral plexus, especially in patients with persistent nerve-related symptoms despite normal or equivocal routine spine or pelvic imaging, or even electromyography. Due to a wide range of compressive and noncompressive neural entities [59], MRN is useful to localize the lesion, to provide direct and noninvasive evidence of neuromuscular pathologies, guiding therapy, and additional studies. MRN can be performed any time after the neurological deficit, as opposed to EMG, which normally require a period of 2 to 3 weeks to detect abnormalities. MRN is a promising method and should be widely understood and performed in daily radiology practice.

## Figures and Tables

**Table 1:** Clinical indications for MRN of the lumbosacral plexus.

To confirm the involvement of the lumbosacral plexus and its extension in patients with tumor diseases
To assess the extent of injuries
To evaluate the lumbosacral plexus in patients with indeterminate MR of the lumbar spine
To exclude presence of masses in patients with unilateral changes in EMG
To exclude lesions in patients with persistent symptoms and normal or indeterminate findings on EMG
To confirm changes of lumbar plexus in patients with confusing clinical findings
To evaluate abnormalities of peripheral nerve branches and associated lesions (e.g., piriformis syndrome, pudendal neuralgia, and meralgia paresthetica)
To plan administration of medications guided by imaging

*Note*. MRN: magnetic resonance neurography; MR: magnetic resonance; EMG: electromyography.

**Table 2:** Summary of anatomic features of the lumbosacral plexus nerves.

Nerve	Origin	Muscles innervated	Sensory distribution
Lumbar plexus			

Anterior rami	L2-3	(i) Psoas	None

Iliohypogastric	T12-L1	(i) Transversus abdominus (ii) Internal oblique	(i) Anteromedial abdominal wall (ii) Lateral buttock

Ilioinguinal	L1	(i) Transversus abdominus (ii) Internal oblique	(i) Medial aspect of the upper thigh (ii) Base of penis and anterior scrotum (iii) Mons pubis and superior labia majora

Genitofemoral	L1-L2	(i) Cremaster (males)	(i) Anterior aspect of the upper thigh. Spermatic cord and posterior scrotum (ii) Inferior labia majora

Femoral	L2–L4	(i) Rectus femoris (ii) Vastus lateralis (iii) Vastus medialis (iv) Sartorius (v) Pectineus (vi) Iliacus	(i) Anterior and medial aspects of the mid and distal thigh (ii) Knee joint

Lateral femoral cutaneous	L1–L3	None	(i) Lateral thigh

Obturator	L2–L4	(i) Obturator externus (ii) Adductor magnus (iii) Adductor longus (iv) Adductor brevis (v) Gracilis (vi) Pectineus	(i) Medial aspect of the upper thigh

Lumbosacral trunk	L4-L5	None	None

Sacral plexus			

Sciatic	L4–S3	(i) Biceps femoris (ii) Semitendinosus (iii) Semimembranosus (iv) Adductor magnus	(i) Leg

Superior gluteal	L4–S1	(i) Gluteus medius (ii) Gluteus minimus (iii) Tensor of the fascia lata	None

Inferior gluteal	L5–S2	(i) Gluteus maximus	None

Pudendal	S2–S4	(i) External anal sphincter (ii) External urinary sphincter	(i) External genitalia

Posterior femoral cutaneous	S1–S3	None	(i) Inferior and medial buttock (ii) Hip joint (iii) Perineum (iv) Popliteal fossa

**Table 3:** Departmental protocol for MRN of the lumbosacral plexus.

Sequence	FOV (cm)	TR/TE (ms)	Slice thickness (mm)

Sagittal STIR	24	3500/18	4
Sagittal T1 fast spin-echo	24	800/12	4
Coronal STIR 3D SPACE	32	1500/100	1
Coronal T2 3D isotropic	32	1500/140	1
Axial T1 fast spin-echo pre- and postcontrast	32	803/12	4
Axial T2 SPAIR	32	4500/79	4
Axial diffusion	32	5800/86	4
Coronal T1 3D VIBE pre- and postcontrast	38	4.39/1.92	1

**Table 4:** Muscle denervation changes in MR imaging.

Phase	Duration	Imaging findings		
		T1	T2	T1C+
Acute	<1 month	Normal	↑	+
Subacute	1–6 months	±Normal	↑	±
Chronic	>6 months	↑	↓	−

**Table 5:** Pitfalls for MRN interpretation.

Magic angle effect
Increased perineural T2 signal due to adjacent vessels
Inhomogeneous fat suppression
3 T MRN increased susceptibility and chemical shift artifacts

**Table 6:** Summary of features for main lesions in MRN.

Etiology	Enlargement	T2 hyperintensity	Fascicular pattern	Course	Enhancement
Trauma	+/−	+	Minimally effaced/disrupted	May be altered due to hematoma/fracture	−

CIDP	+/−	++	Effaced/preserved	Not altered	++

Infectious	+/++	++	Effaced/preserved	May be altered by abscess/granulation tissue	++

CMT	++	++	Preserved or fatty infiltration around atrophic fascicles	Normal	+/−

Radiation	+/− (linear geographic distribution of radiation field)	+	Preserved/effaced	Altered in subacute-chronic stages due to developing fibrosis	+

NF1	++	++	Preserved	Altered with focal masses	+/++
